# Impact of blood levels of progesterone on the day of ovulation onset on clinical, laboratory and reproductive parameters of young patients undergoing assisted reproduction: a cross-sectional study

**DOI:** 10.31744/einstein_journal/2022AO6896

**Published:** 2022-05-16

**Authors:** Renata Garcia Olmos Fernandez, Caio Parente Barbosa, Denise Maria Christofolini, Luiz Vinicius de Alcantara Sousa, Renato de Oliveira, Bianca Bianco

**Affiliations:** 1 Centro Universitário FMABC Santo André SP Brazil Centro Universitário FMABC, Santo André, SP, Brazil.

**Keywords:** Chorionic gonadotropin, Fertilization *in vitro*, Infertility, Progesterone

## Abstract

**Objective:**

To assess whether there is an association between the level of progesterone on the day of administration of human chorionic gonadotropin and clinical and laboratory characteristics, in addition to the results of in vitro fertilization of patients with a good prognosis.

**Methods:**

A cross-sectional study comprising 103 women who underwent intracytoplasmic sperm injection treatment, between November 2009 and May 2015, aged ≤35 years, with no comorbidities, with fresh embryo transfer. Data were collected from patient medical records.

**Results:**

There was a weak positive correlation between the level of progesterone on the day of human chorionic gonadotropin and the number of follicles larger than 14mm (ß=0.02, p=0.001), retrieved oocytes (ß=0.01, p=0.01) and oocytes in metaphase II (MII) (ß=0.02, p=0.02); that is, the increase in progesterone level has a slight association with increased values of these variables. Body mass index was inversely correlated with progesterone level on the day of human chorionic gonadotropin (ß=-0.01, p=0.02). No association was found between the level of progesterone on the day of human chorionic gonadotropin and the protocols used for controlled ovarian stimulation, quality of transferred embryos and the pregnancy rate.

**Conclusion:**

There is an association between the value of progesterone on the day of human chorionic gonadotropin administration with body mass index, number of follicles larger than 14mm, number of retrieved oocytes and oocytes in metaphase II. Unlike embryo quality and pregnancy rate, which do not have a statistically significant relation with this value in the population studied.

## INTRODUCTION

Since the first birth by *in vitro* fertilization (IVF), in 1978, several clinical, endocrine and laboratory hypotheses have been raised to improve the performance of the method and predict the negative variables that accompany the therapeutic process, both in the study of the female body and of the embryo.^(1)^

Recently, there have been more endocrinological studies to assess the progesterone level on the day when oocyte maturation ends with the use of human chorionic gonadotropin (hCG) or gonadotropin-releasing hormone (GnRH); and its possible interference in the results of IVF.^(2)^ Progesterone is produced by the corpus luteum from the moment ovulation occurs, approximately 24 hours after the peak of luteinizing hormone (LH), when the luteal phase of the menstrual cycle begins. Progesterone acts on the endometrium and changes its characteristics from proliferative to secretory.^(3)^

In the IVF cycle induced with supraphysiological doses of exogenous follicle stimulating hormone (FSH), premature luteinization (term used for increase in progesterone in the late follicular phase) often occurs, despite the use of GnRH agonists and antagonists for hypothalamic block. A progesterone level >1.0ng/mL is already considered for premature luteinization.^(4)^ This could be explained by the stimulation of growth of multiple follicles and high production of estradiol, which would stimulate the granulosa cells to produce insufficient amounts of progesterone for ovulation, but acting on the endometrium. In this way, the endometrium becomes secretory prematurely, changing the date of the embryonic implantation window and making it unpredictable.^(4)^

In 2015, Chen et al. showed through endometrial biopsy that women with progesterone levels ≥1.7ng/mL, on the day of hCG application, had endometrium with altered expression of angiogenic factors from the vascular endothelial growth factor (VEGF) family on the seventh day after hCG, which decreased the implantation rate due to reduced endometrial receptivity.^(5)^

In this sense, the progesterone dosage on the day of the onset of final oocyte maturation may be a predictor of endometrial synchronization in relation to the embryo, which may help in the clinical management with cancellation of fresh transfer. However, there is still controversy in the literature on the subject, mainly due to the fact that early luteinization is frequent, occurring in 5%-50% of cases. Therefore, adopting a cut-off value for progesterone levels on the day of hCG may increase the rate of frozen embryos and patients without fresh embryo transfer.^(6)^

Some studies, including a recently performed multicenter investigation^(7)^ and a meta-analysis of over 60,000 IVF cycles,^(8)^ considered the progesterone level on the day of hCG is important and should be valued. The latter considered progesterone levels ≥0.8ng/mL on the trigger day decreased the IVF implantation rate by approximately 10%.^(8)^ However, cut-off values for progesterone varied in different studies from 0.4 to 1.7ng/mL.^(9)^ In addition, Oktem et al. determined the progesterone cut-off value could change depending on the response to controlled ovarian stimulation.^(10)^

Furthermore, most studies do not exclude other factors that may be involved with negative beta human chorionic gonadotropin (βhCG) fraction after IVF, such as age of the woman, premature ovarian failure, obesity, polycystic ovary syndrome, endometriosis, ovarian and/or or uterine surgery, body mass index (BMI), smoking, use of drugs, severe male factor requiring an invasive procedure for sperm capture, and poor quality embryo transfer,^(11)^ which makes it difficult to interpret the real value of the progesterone level on the day of hCG.

Thus, a more careful selection of patient characteristics such as BMI ≤30kg/m^2^, hormone levels within normal limits, age ≤35 years, and absence of comorbidities would help to minimize this variation of values demonstrated in the literature.

## OBJECTIVE

To assess whether there is an association between the level of progesterone on the day of administration of the human chorionic gonadotropin and the clinical and laboratory characteristics, in addition to the results of *in vitro* fertilization of patients with a good prognosis.

## METHODS

A cross-sectional study including 103 women who underwent intracytoplasmic sperm injection (ICSI) treatment at *Instituto Ideia Fértil, Faculdade de Medicina do ABC,* Santo André, SP, Brazil, between November 2009 and May 2015.

### Ethical aspects

Clinical and hormonal data, and reproductive results were collected after exposing the study objectives in the Informed Consent Form, approved by the Research Ethics Committee of the *Centro Universitário FMABC,* (# 676628, CAAE: 31010214.3.0000.0082).

Data such as age, BMI, FSH, prolactin, cause of infertility, antral follicle count (AFC), protocol and days of controlled ovarian stimulation (COS), ovarian response to COS, number of follicles visualized on ultrasound, oocytes retrieved, oocytes in metaphase II (MII), embryos and pregnancy were collected from the medical records of the study participants.

### Patients

A total of 1,159 medical records were searched and included only women aged ≤35 years, who underwent ICSI, with serum levels of thyroid-stimulating hormone (TSH) (0.5-4.0IU/L) and prolactin (≤30.0ng/mL) within the parameters of normality, presence of both ovaries with no morphological abnormalities, ovulatory cycles lasting 21 to 35 days, BMI ≥18.5 and ≤30kg/m^2^, without evidence of endocrine diseases, who evaluated the level of progesterone on the day of hCG administration, and who have transferred one or two embryos, according to common practice of the service, and respecting the resolution of the Federal Council of Medicine of 2017.^(12)^ We also included women whose partner’s semen analysis was abnormal, since this factor does not interfere with the ovarian response.

Exclusion criteria comprised smoking, drug use, endometriosis (diagnosed by videolaparoscopy/laparotomy and histological evidence of the lesions), hydrosalpinx, premature ovarian failure, plasma progesterone >1.7ng/mL (patients without fresh embryo transfer due to the service cut-off value), trigger done with GnRH agonist, uterine malformations, number of oocytes retrieved <3, and ovarian hyperstimulation syndrome (OHSS) with subsequent embryo freezing. Only one case of OHSS was included, since it had only 12 MII oocytes and consequent fresh embryo transfer. Thus, out of the 1,159 medical records analyzed, after observing the inclusion and exclusion criteria, 1,056 cases were excluded, and 103 patients were included in the study. Progesterone of all patients was measured in the organization laboratory, in the morning of the day the trigger was performed, thus avoiding bias in the comparison of dosages.

The investigation into the cause of infertility at the service was carried out in accordance with basic workup, following the guidelines of the American College of Obstetricians and Gynecologists (ACOG) and the American Society of Reproductive Medicine (ASRM), and included a comprehensive medical history, physical examination, hormone and biochemical profile, testing for sexually transmitted infections, imaging tests (transvaginal ultrasound, hysterosalpingography) and semen analysis.^(13,14)^ Additionally, as a routine of this service, patients are also requested to have undergone diagnostic hysteroscopy to rule out polyps, fibroids, and malformations in the uterine cavity.

The tubal integrity was analyzed by hysterosalpingography and/or laparoscopy. The anatomical alterations of the fallopian tubes that impede their proper functioning, such as tubal obstructions, functional alterations caused by, *e.g.*, pelvic inflammatory disease (PID) or previous tubal surgeries were considered as tubal-peritoneal factor. In the absence of changes in this assessment, infertility was considered idiopathic.

According to the World Health Organization (WHO) criteria, the male factor was considered when there was an initial concentration of spermatozoa lower than 15million/mL, less than 40% of motile sperm, considering both progressive and non-progressive sperm, or asthenozoospermia with less than 32%, if only fast and progressive sperm are considered.^(15)^

### Antral follicle count

Before the start of COS, on the first to third day of the menstrual cycle, patients were submitted to AFC by conventional two-dimensional transvaginal ultrasound, at a frequency of 7MHz (Philips^®^). Antral follicles were those with a maximum diameter of 10mm.^(16)^

### *In vitro* fertilization treatment

The studied patients underwent COS with daily fixed doses of 100, 150 or 200IU of recombinant FSH (rFSH), for 8 to 14 days, according to institutional protocols. To block LH peak, in the protocol with the use of a GnRH agonist, leuprorelin acetate 3.75mg (Lupron^®^, Abbott Laboratories, Chicago, USA, intramuscular) was used in the luteal phase, prior to the start of treatment. In the variable protocol regarding the introduction of the GnRH antagonist, which we call “antagonist”, when the largest follicle reached 14mm, daily GnRH antagonist 0.25mg (Orgalutran^®^, Schering-Plough, Kenilworth, USA, subcutaneous absorption) was introduced and maintained together with rFSH until the follicles achieved between 17mm and 20mm in diameter, with monitoring by transvaginal ultrasound. At this moment, 5000IU hCG (Choriomon^®^, IBSA Institut Biochimique SA, Lamone, Switzerland, subcutaneous absorption) was used for the trigger and peripheral blood was collected by venipuncture to measure plasma progesterone. Ovarian puncture occurred 35 hours after trigger and, on the same day, support of the luteal phase with vaginal micronized progesterone was started (600mg per day, single daily dose).

Patients were divided into two groups, considering the number of follicles larger than 14 mm, after a minimum period of seven days of COS with gonadotropins: “low response” women who had ≤3 follicles larger than 14mm and “satisfactory response” ≥4 follicles larger than 14mm.

Metaphase II fertilization was performed using the ICSI technique, in all cases, according to the routine of the service. Embryos were cultured for 3 to 6 days, and a maximum of two embryos were transferred at D3 or D5, depending on the age of the woman and the cause of infertility. Pregnancy was confirmed by measuring βhCG (>25mIU/mL) on the 12^th^ day after embryo transfer.

### Statistical analysis

Absolute and relative values were used to describe the qualitative variables; for quantitative variables that did not show normal distribution according to the Shapiro-Wilk test, we used median and 95% confidence interval (95%CI).

We used linear regression and Spearman’s correlation to assess the association of progesterone levels with the variables age, BMI, FSH, LH, TSH, prolactin, blood glucose, AFC, duration and total dose of rFSH used in COS, number of follicles larger than 14mm, MII, and number of embryos obtained. The Kruskal-Wallis and Mann-Whitney tests were used to assess the association of progesterone levels with the cause of infertility, semen analysis, type of protocol used for COS, and quality of transferred embryos. The *χ*^2^ test was used to assess the association of progesterone levels and pregnancy rate. Pearson’s correction was used to assess the association between BMI and levels of progesterone and oocytes, according to the response to COS. We used 95%CI for all analyses. The program used was Stata version 16.0.

## RESULTS

The clinical characteristics, hormone profile and reproductive outcomes of the women studied are shown in table 1.

**Table 1:** Clinical characteristics, hormone profile and reproductive outcomes of the women studied

Variables		Median (95%CI)
n		103
Age, years*		33 (32-33)
BMI (kg/m^2^)*		24.4 (23.1-25.0)
Infertility type, n (%)		
	Primary	77 (74.8)
	Secondary	26 (25.2)
Cause of infertility, n (%)		
	Male factor	79 (76.7)
	Tubal factor	4 (3.9)
	Male factor + tubal factor	12 (11.6)
	Idiopathic	8 (7.8)
TSH (IU/mL)*		1.8 (1.50-1.90)
LH (mIU/mL)*		4.9 (4.5-5.1)
FSH (IU/mL)*		6.6 (6.3-6.9)
Prolactin (ng/mL)*		14 (13.0-16.0)
AFC*		10 (10-11)
COS protocol, n (%)		
	Antagonist 100IU	34 (33.0)
	Antagonist 150IU	20 (19.4)
	Antagonist 200IU	48 (46.6)
	Agonist 100IU	1 (1.0)
COS days (days)*		9 (9.0-9.4)
Total dose of rFSH*		1,500 (1,350-1,650)
Progesterone in hCG day (ng/mL)*		0.7 (0.6-0.8)
Follicles >14mm*		8 (7-8)
Retrieved oocytes*		7 (6-7)
MII*		6 (4.1-6.0)
Embryos*		3 (2-3)
Transferred embryos*		2 (2-2)
Quality of transferred embryos, n (%)		
	Absence of embryos	3 (2.9)
	D3	75 (72.8)
	D5	25 (24.3)
Pregnancy/cycle, n (%)		50 (48.5)

* variables were presented as median and 95% confidence interval.

BMI: body mass index; TSH: thyroid-stimulating hormone; LH: luteinizing hormone; FSH: follicle stimulating hormone; AFC: antral follicle count; COS: controlled ovarian stimulation; rFSH: recombinant follicle stimulating hormone; hCG: human chorionic gonadotropin; MII: oocytes in metaphase II; D3: days 3; D5: days 5.

Considering the semen analysis pattern, 17.5% (18/103) of cases did not present changes in relation to the concentration per mL; 14.6% (15/103) had mild oligozoospermia; 27.2% (28/103) moderate oligozoospermia; 18.5% (19/103) severe oligozoospermia; 15.5% (16/103) had obstructive azoospermia and were subjected to percutaneous epididymal sperm aspiration (PESA); and 6.8% (7/103) used donor semen.

Table 2 shows the correlation between the level of progesterone on the day of hCG and the clinical, hormone and reproductive characteristics of the women studied. According to the results presented, there was a weak positive correlation between the level of progesterone on the day of hCG and the number of follicles larger than 14mm, retrieved oocytes and MII. This means that, as the level of progesterone increases, these variables also slightly increase. Body mass index was inversely correlated with progesterone level on the day of hCG.

**Table 2:** Correlation between progesterone level on the day of administration of human chorionic gonadotropin and the clinical, hormone and reproductive characteristics of the women studied

Variables	β	p value*
Age	-0.01	0.267
BMI	-0.01	0.02
Type of infertility	0.32	0.14†
FSH	0.01	0.4
LH	0.01	0.39
TSH	-0.015	0.59
Prolactin	0.006	0.06
Blood glucose	0.003	0.378
AFC	-0.004	0.53
COS duration	-0.006	0.76
Total dose of rFSH	0	0.32
Visualized follicles	0.02	0.001
Retrieved oocytes	0.01	0.01
MII	0.02	0.02
Embryos	0.015	0.297

* Spearman correlation;

† Kruskal-Wallis test.

BMI: body mass index; FSH: follicle stimulating hormone; LH: luteinizing hormone; TSH: thyroid-stimulating hormone; AFC: antral follicle count; rFSH: recombinant follicle stimulating hormone; MII: oocytes in metaphase II.

Table 3 shows the relation between the level of progesterone on the day of hCG and the protocols used for COS, the quality of transferred embryos and pregnancy. The results showed no association between the level of progesterone on the day of hCG and the research variables.

**Table 3:** Relation of progesterone level on the day of human chorionic gonadotropin according to the types of protocols used for controlled ovarian stimulation in *in vitro* fertilization treatments, embryonic quality, and pregnancy

Variables		Progesterone (ng/mL)*	p value
COS protocols			0.40†
	Antagonist 100IU	0.65 (0.5-0.8)	
	Antagonist 150IU	0.6 (0.51-0.72)	
	Antagonist 200IU	0.8 (0.6-0.9)	
	Agonist 100IU	0.6 (0.6-0.6)	
Embryo quality			
	Absence of embryos	0.67 (0.33-0.8)	0.74†
	D3	0.73 (0.59-0.8)	
	D5	0.7 (0.6-0.8)	
Pregnancy			
	Positive	0.7 (0.59-0.77)	0.477#
	Negative	0.73 (0.59-0.9)	

* progesterone level was presented as median and 95% confidence interval;

† Kruskal-Wallis test;

# Mann-Whitney.

COS: controlled ovarian stimulation; D3: day 3; D5: day 5.

Table 4 shows the clinical characteristics, hormone profile and reproductive results of the women studied, according to the COS response. Women with >14 follicles were subjected to trigger with a GnRH agonist due to the possible risk of OHSS and subsequent embryo freezing, and did not participate in the study. However, the only case of a patient in the “satisfactory response” group with 15 follicles larger than 14mm was included due to aspiration of 12 MII oocytes and fresh embryo transfer (hCG was performed for trigger).

**Table 4:** Reproductive outcomes results of the women studied, according to the controlled ovarian stimulation

Variables		Response to controlled ovarian stimulation		p value
		Low	Satisfactory	
n		13	90	
Age, years		33 (32.4-34)	33 (32-33)	0.302†
BMI (kg/m^2^)		26.9 (24.73-30.11)	24.0 (22.53-24.88)	0.017†
Infertility type (%)				
	Primary	76.92	74.44	0.045#
	Secondary	23.08	25.56	
TSH (IU/mL)*		1.7 (1.33-2.45)	1.8 (1.5-1.9)	0.988†
LH (mIU/mL)*		4.7 (3.39-5.50)	4.95 (4.43-5.28)	0.413†
FSH (IU/mL)*		6.4 (5.97-7.14)	6.7 (6.3-6.98)	0.736†
Prolactin (ng/mL)*		14 (13.23-17.45)	13.9 (11.41-16.36)	0.644†
AFC*		11 (5.78-12)	10 (10-11)	0.531†
COS days (days)*		9 (8-10)	9 (9-10)	0.588†
Total dose of rFSH*		1,350 (1,050-1,660)	1,500 (1,350-1,744)	0.132†
Progesterone on the day of hCG (ng/mL)*		0.5 (0.33-0.82)	0.73 (0.6-0.8)	0.036†
Oocytes *		2 (2-3)	7 (7-8)	<0.001†
MII*		2 (1-3)	6 (5-7)	<0.001†
Embryos*		2 (1-2)	3 (2-3)	0.001†
Transferred embryos		2 (1-2)	2 (2-2)	0.491†
Quality of transferred embryos, n (%)				
	Absence of embryos	1 (7.69)	3 (3.33)	0.105&
	D3	12 (92.31)	62 (68.89)	
	D5	0	25 (27.78)	
Pregnancy/cycle, n (%)		7 (53.85)	43 (47.78)	0.710#

* variables were presented as median and 95%CI.

† Kruskal-Wallis test;

# *χ*^2^ Test;

& Mann-Whitney test.

BMI: body mass index; TSH: thyroid-stimulating hormone; LH: luteinizing hormone; FSH: follicle stimulating hormone; AFC: antral follicle count; COS: controlled ovarian stimulation; rFSH: recombinant follicle stimulating hormone; hCG: human chorionic gonadotropin; MII: oocytes in metaphase II; D3: day 3; D5: day 5.

Furthermore, the greater the response to COS, the mean progesterone value also increased (0.5 and 0.73 in the low and satisfactory response groups, respectively). In table 2, the higher the progesterone, the greater the number of follicles >14mm. The level of progesterone on the day of hCG was significantly different between women who had a low response and a satisfactory response to COS (0.5ng/mL and 0.73ng/mL, respectively, p=0.03). Body mass index, oocytes, MII, number of embryos and transferred embryos also showed a statistically significant difference when comparing these two groups (p=0.01 for BMI, and p<0.001 for other variables).

Body mass index was also analyzed in relation to the number of oocytes and progesterone, to verify whether there could be a confusing factor, that is, if these patients could have a lower serum progesterone value on the day of hCG because they had fewer oocytes and less ovarian response. We observed an inversely proportional correlation between BMI and progesterone levels (r=-0.22 and p=0.029) and a directly proportional correlation considering the number of oocytes (r=0.25 and p=0.011).

Table 5 shows both patients with D3 and D5 transferred embryos who did not get pregnant had a higher median progesterone than those who got pregnant. When comparing only the group of transferred blastocysts, the progesterone value in the group of women with a positive biochemical pregnancy is lower than that in the group of women with a negative biochemical pregnancy.

**Table 5:** Progesterone value in relation to embryonic quality according to pregnancy outcome (progesterone level was presented as median)

Embryo quality	Biochemical pregnancy		p value*
	Positive	Negative	
D3	0.71 (0.56-0.8)	0.73 (0.52-0.9)	0.404
D5	0.60 (0.50-0.81)	0.80 (0.47-0.97)	

* *χ*^²^ test.

D3: day 3; D5: day 5.

## DISCUSSION

The influence of progesterone on IVF/ICSI cycles has been discussed for some years and, so far, there is no consensus on the obligation to measure its plasma value on the day of hCG administration to decide whether or not to perform embryo transfer, nor what would be its cut-off value, based on the premise that it must be dosed.^(17)^ Although many centers empirically adopt the cut-off value of 1.5ng/mL, there is a wide spectrum of variation, which encompasses more than 100% of difference between the lowest and highest values adopted. In addition to the potential impact of progesterone on the endometrium, other authors suggest the possibility of impaired ovarian response to COS, oocyte maturation, fertilization, and embryonic cleavage due to premature luteinization. Thus, despite the numerous studies, there is still much to be researched on the subject.^(6)^

Oktem et al. and Xu et al. highlighted that there would be an associated progesterone value for a cut-off score for each type of ovarian response - hyporesponders, normoresponders or hyperresponders - and, therefore, we could not use a universal cut-off value for all IVF cases to evaluate the possibility of fresh embryo transfer.^(10,18)^ Kyrou et al. showed estradiol level greater than 1790.5pg/mL, associated with more than 10 follicles with at least 11mm in diameter on the day of hCG administration, has a strong correlation with progesterone levels greater than 1.5ng/mL;^(19)^ These results agree with those obtained in our study, in which a greater number of pre-ovulatory follicles was associated with a greater production of progesterone. Consequently, with a greater number of follicles, there is also a greater number of retrieved and MII oocytes. Thus, the increase in progesterone on the day of hCG from the greater number of retrieved oocytes does not seem to affect the quality of oocyte maturation in the studied group of young patients. In 1997, Ubaldi et al. had already shown, through endometrial biopsy of patients with serum progesterone levels greater than 1.1ng/mL, that this condition reflected an asynchronous endometrium in relation to the embryo (three days advanced), which negatively affected the rate of implantation and pregnancy.^(20)^ In 2017, Xiong et al. published that progesterone values above 1.5ng/mL change endometrial receptivity because they are associated with histological advancement in the endometrium, which can correspond to more than three days of anticipation; in addition to abnormal expression of implantation regulatory proteins - VEGF and placental growth factor (PGF); abnormalities in gene expression that may be related to natural killer (NK) cells activity and differences in epigenetic profiles.^(21)^ However, the strategy of freezing embryos for later transfer after endometrial preparation, which would be the solution for cases of high progesterone, increases the cost of treatment and, as a result, patient themselves often prefer fresh transfer.^(22)^

Some authors report that high progesterone would occur due to the exacerbated response to stimulation of ovaries.^(8,23)^ Therefore, cycles with lower doses of rFSH could avoid this condition, despite the higher risk of fewer oocytes retrieved, and consequently, fewer embryos and a lower pregnancy rate.^(24)^ In contrast, studies showed that high progesterone did not change the pregnancy rate in cases of hyperresponders.^(17)^ This raises the question whether a better response to COS, with more follicles and more oocytes, would lead to a greater number of better quality embryos and a higher implantation rate, even with an endometrium potentially affected by the increase in progesterone levels or, whether a lower response to COS, with a lower number of embryos, would lead to a lower implantation rate, which would still worsen with high progesterone.^(25)^

Luteinizing hormone plays a central role in inducing progesterone secretion by the ovaries. However, evidence suggests that progesterone biosynthesis can also be regulated by local factors that exert their effects in an autocrine and/or paracrine manner, such as the adrenals.^(26)^

Considering the embryo quality, there is evidence about its alteration due to rise in progesterone; that is, the rate of formation of high quality embryos decreases as progesterone increases.^(27)^ However, Fanchin et al., Yang et al., Bosch et al., and Papanikolaou et al. corroborate our study and observed increased progesterone would not alter the quality of oocytes or embryos.^(25,28-30)^ Furthermore, Papanikolaou et al. hypothesized only in cases of embryo transfer at D3 there would be interference from progesterone – if progesterone >1.5ng/mL on the day of hCG, the implantation rate would drop by 50%; the same would not occur in the transfer of blastocysts, because in these cases the endometrium would have recovered sufficiently to allow a better adequacy between the embryo and the endometrium, enhancing implantation.^(30)^

Instead, Hill et al. showed patients with a good prognosis and blastocyst transfer, are also negatively affected by their results, regardless of the ovarian response, as long as progesterone is increased on the day of hCG (progesterone >1.5 to 2.0ng/mL).^(31)^ This study observed higher progesterone would be associated with a decrease in the live birth rate - independent of embryonic stage, age, embryonic quality and COS. In addition, it would not be associated with a higher number of oocytes, unlike in our study. However, they included patients of all ages and adjusted the results for the selected group, instead of including only patients with a good prognosis, according to the inclusion criteria of the present study. In addition, there was no association with pregnancy rate in our study, since the protocol empirically adopted at the patient’s treatment site recommended no embryo transfer in cases of progesterone values higher than 1.7ng/mL. As the median progesterone in our study was 0.7ng/mL, this fact may also explain why progesterone does not impact pregnancy outcomes.

We observed BMI was significantly higher in women who had a low response to COS compared to those who had a satisfactory response [26.9 (24.73-30.11) *versus* 24.0 (22.53-24.88), p=0.017]. However, BMI was inversely proportional to progesterone levels (r=-0.22 and p=0.029) and directly proportional considering the number of oocytes (r=0.25 and p=0.011). Thus, it is possible that the higher the BMI, the higher the aromatase expression and, consequently, the higher the estrogen production, which could impact the production of progesterone.^(32)^ In addition, obesity and higher BMI, with a higher degree of adiposity, can affect the pharmacokinetic and pharmacodynamic processes of hormones. This explains the fact that equal doses of emergency contraceptives and contraceptive patches, *e.g.,* are less effective in women with high BMI compared to adequate BMI, and could also explain progesterone inversely correlated with BMI.^(33)^

Rochester et al. observed obese nonpregnant women had approximately 75%-80% lower luteal progesterone levels than normal weight women.^(34)^ Whynott et al. concluded in their study that body weight was a significant factor that affected the serum progesterone level at the time of pregnancy testing after a cycle of cryopreserved embryo transfer.^(35)^ Friedler et al. evaluated the influence of BMI on the results of 1,654 IVF/ICSI cycles, which were divided into four groups according to the patients’ BMI: group I (normal weight, BMI <25) (943 cycles); group II (overweight, BMI 25-30) (403 cycles); group III (obese, BMI 30-35) (212 cycles), and group IV (morbid obesity, BMI >35) (96 cycles). Response to COS was comparable between groups in terms of mean estradiol and progesterone levels on the day of hCG administration, mean number of oocytes retrieved, fertilized, and number of embryos transferred. Endometrial thickness was significantly smaller in group IV. However, implantation rate, pregnancy per cycle and per transfer, as well as live birth rates did not differ significantly between groups.^(36)^ It appears that the impact of obesity is gradual, being more significant in severe obesity.

### Limitations of the study

The retrospective nature brings limitations to the study. In addition to the small sample size, different doses of rFSH were included in the COS protocols. Furthermore, the heterogeneity of patients and the different causes of infertility can also impact the results. It is noteworthy that infertility without an apparent cause does not mean the absence of causes of infertility, but no identification with the methods and instruments used. However, we included young women without comorbidities and without apparent ovarian changes; in this way, within the instruments used to assess infertility, the effects of the group’s heterogeneity were minimized.

In our study, to comply with the inclusion criteria and select women without comorbidities and ovarian alterations, which could interfere with the quality of the oocytes, we ended up including a large number of cases with male factor and tubal factors, which do not affect implantation (impervious and/or fixed tubes). Thus, it is worth mentioning that pregnancy and implantation rates may vary simply because the embryos are of inferior quality due to the male factor, and not due to the value of progesterone. The preponderance of the male factor would justify a possible impact on reproductive outcomes due to possible direct interference in embryonic quality and implantation. However, this would not interfere with the primary outcomes of the study, which include only the responses related to the female part (progesterone value, number of follicles, retrieved oocytes and mature oocytes).

Finally, as the median progesterone in our study was 0.7ng/mL, this fact may also explain why this hormone does not impact pregnancy outcomes. However, the study does not primarily aim to assess the clinical results of implantation, but rather what are the probable independent variables that could influence an extrapolated and early increase in progesterone, to aid in the most appropriate clinical management.

## CONCLUSION

In the group of patients with good reproductive prognosis studied, there was a directly proportional correlation between the value of progesterone and the number of follicles larger than 14mm, number of oocytes and oocytes in metaphase II, and as the former increases, so does the value of progesterone.
